# Do supervised weekly exercise programs maintain functional exercise capacity and quality of life, twelve months after pulmonary rehabilitation in COPD?

**DOI:** 10.1186/1471-2466-7-7

**Published:** 2007-05-16

**Authors:** Lissa M Spencer, Jennifer A Alison, Zoe J McKeough

**Affiliations:** 1 Physiotherapy Dept, Royal Prince Alfred Hospital, Sydney, Australia; 2 Discipline of Physiotherapy, University of Sydney, Sydney, Australia; 3 Dept Respiratory Medicine, Royal Prince Alfred Hospital, Sydney, Australia

## Abstract

**Background:**

Pulmonary rehabilitation programs have been shown to increase functional exercise capacity and quality of life in COPD patients. However, following the completion of pulmonary rehabilitation the benefits begin to decline unless the program is of longer duration or ongoing maintenance exercise is followed. Therefore, the aim of this study is to determine if supervised, weekly, hospital-based exercise compared to home exercise will maintain the benefits gained from an eight-week pulmonary rehabilitation program in COPD subjects to twelve months.

**Methods:**

Following completion of an eight-week pulmonary rehabilitation program, COPD subjects will be recruited and randomised (using concealed allocation in numbered envelopes) into either the maintenance exercise group (supervised, weekly, hospital-based exercise) or the control group (unsupervised home exercise) and followed for twelve months. Measurements will be taken at baseline (post an eight-week pulmonary rehabilitation program), three, six and twelve months. The exercise measurements will include two six-minute walk tests, two incremental shuttle walk tests, and two endurance shuttle walk tests. Oxygen saturation, heart rate and dyspnoea will be monitored during all these tests. Quality of life will be measured using the St George's Respiratory Questionnaire and the Hospital Anxiety and Depression Scale. Participants will be excluded if they require supplemental oxygen or have neurological or musculoskeletal co-morbidities that will prevent them from exercising independently.

**Discussion:**

Pulmonary rehabilitation plays an important part in the management of COPD and the results from this study will help determine if supervised, weekly, hospital-based exercise can successfully maintain functional exercise capacity and quality of life following an eight-week pulmonary rehabilitation program in COPD subjects in Australia.

## Background

Chronic Obstructive Pulmonary Disease (COPD) is the fourth leading cause of death in Australian males, the sixth leading cause of death in females [1] and resulted in 5000 deaths in 2003 [2]. COPD is a progressive, disabling disease, which has a significant impact on the health care system [1,3]. Early diagnosis and management of COPD is a priority of health care services throughout Australia. Management of COPD involves optimising medical therapy, commencing smoking cessation and participating in pulmonary rehabilitation.

Pulmonary Rehabilitation is a multi-disciplinary approach to the management of patients with respiratory disease. The program is individually tailored to the needs of the patient, combining exercise, self-management education and psychosocial support [4]. Pulmonary rehabilitation has been shown to improve functional exercise capacity, quality of life and dyspnoea, and to reduce hospital admissions and length of stay [5].

Following pulmonary rehabilitation, benefits in terms of functional exercise capacity and quality of life are maintained for up to nine months [6-8]. However, the benefits appear to decline by twelve months [9] unless the program is of longer duration [10] or involves ongoing maintenance exercise [11,12]. There is little evidence to support the best method of maintaining these gains long-term and the above studies differ in length and intensity of the exercise programs. As the majority of pulmonary rehabilitation programs in Australia are of eight weeks duration, it is important to determine the best method of maintaining functional exercise capacity and quality of life, twelve months following an eight-week pulmonary rehabilitation program.

The aim of the study is to determine if supervised, weekly exercise following an eight-week pulmonary rehabilitation program will maintain the benefits to twelve months.

## Methods

### Participants

This study will be a prospective, randomised controlled trial in participants diagnosed with COPD. Participants will be recruited consecutively on completion of an eight-week pulmonary rehabilitation program and randomised into either a maintenance exercise group (ME) or a control group and followed for 12 months. Randomisation will be by computerised random number generation in concealed envelopes prepared by an investigator not involved in subject testing or training. The chief investigator will conduct both testing and training so will be aware of group allocation.

**Figure 1:**
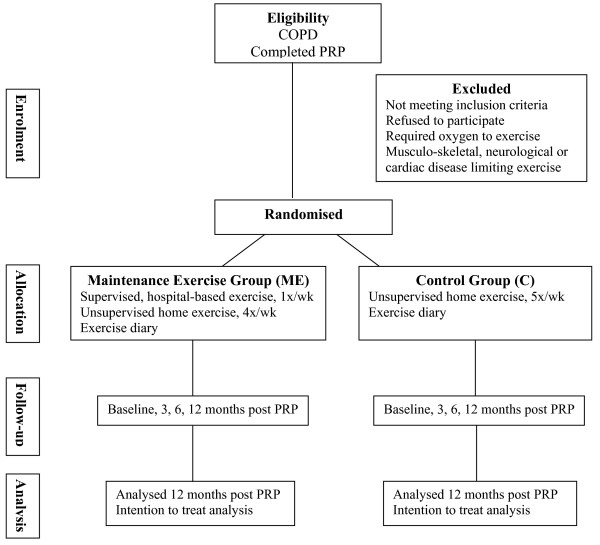
**Flow of participants through the study**. PRP = pulmonary rehabilitation program; COPD = chronic obstructive pulmonary disease; x/wk = times per week.

Participants will be excluded if they have experienced an exacerbation of COPD in the previous month, if they require supplemental oxygen, or if they have co-morbidities such as severe cardiovascular, neurological or musculoskeletal conditions that will prevent them performing functional exercise tests. The flow of participants through the study will reflect the recommendations from the Consolidated Standards of Reporting Trials (CONSORT) statement [13], and is illustrated in Figure 1. Participants will receive written and verbal information explaining the study and written consent will be obtained from all subjects. Ethics approval to conduct the study has been obtained from the Ethics Committee of Sydney South West Area Health Service (Eastern Zone). The study will take place at Royal Prince Alfred Hospital (RPAH), Sydney, Australia.

### Interventions

Participants randomised to the ME group will attend hospital-based pulmonary rehabilitation once a week for supervised exercise training and will also be asked to follow an unsupervised home exercise program on four other days a week. The hospital-based program will be individually designed and supervised by a qualified physiotherapist and will consist of lower limb and upper limb exercise (Table 1) similar to those that the subject performed during the eight-week pulmonary rehabilitation program. More specifically, the program will consist of a) lower limb endurance training in which the participant will perform 20 minutes of stationary cycling and 20 minutes of walking, b) lower limb strength training using weight machines for the quadriceps and gluteal muscles, c) upper limb endurance training which will consist of arm cycle ergometry and unsupported arm exercises with light weights [14], d) upper limb strength training using weight machines for the latissimus dorsi and pectoralis major/minor muscles. All strength-training exercises will be performed in three sets of ten repetitions.

**Table 1:** Components of the exercise programs

	**Supervised**	**Unsupervised**
**Equipment**	Yes	No
**Intensity**	Dyspnoea 3–4 Borg Scale	Dyspnoea 3–4 Borg Scale
**Duration**	20 mins W 20 mins SC 30 mins AC, LWM, AWM, UAE	30 mins W 30 mins S, Sq, S/S, UAE
**Total duration**	70 mins	60 mins
**Frequency** (per wk)	1	4 (ME group) 5 (Control group)
**Venue**	Hospital gym	Home

AC = arm crank ergometer; AWM = arm weight machine; LWM = leg weight machine; ME = maintenance exercise; mins = minutes; SC = stationary cycle; S = step-ups; Sq = wall squats; S/S = sit to stand exercises; UAE = upper limb unsupported arm exercises as described by Ries [14]; W = walking.

Participants randomised to the control group will follow standard care and be asked to follow an unsupervised home exercise program on five days per week which will consist of 30 minutes of walking, unsupported arm exercises [14] and lower limb strengthening exercises five days per week (Table 1). The exercises will be explained and illustrated in the home exercise booklet that all participants will have received during pulmonary rehabilitation. Participants in the ME group will be asked to follow the same home exercise program four days a week. Participants in the control group will not be contacted between testing sessions. Participants in both the ME and control groups will be asked to complete an exercise diary by ticking boxes to indicate the exercises they complete.

### Outcomes measures

**Figure 2:**
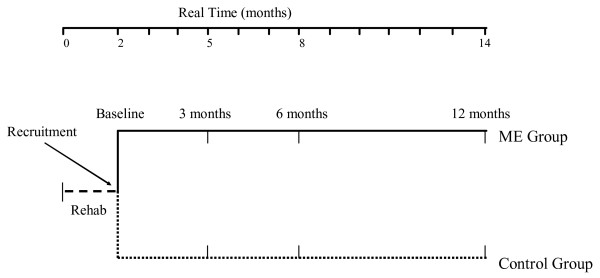
**Timeline for recruitment and measurement**. Rehab = eight week pulmonary rehabilitation program; ME = maintenance exercise.

All outcome measures will be recorded at baseline, three, six and 12 months post pulmonary rehabilitation (Figure 2). The outcome measures are outlined below.

1. Functional exercise capacity will be measured by:

a) Six-minute walk test (6MWT) [15]. This test measures the distance a participant is able to walk in six minutes and will be performed using a protocol standardised for factors known to affect results e.g. instructions, encouragement, number of tests and type of track [15]. Participants will be asked to walk as far as they can in six minutes, to do the best they can, and to cover as much ground as possible. Every minute participants will be made aware of the time and will be given standardised encouragement e.g. "You are doing well – you have 5 minutes to go!" This will be alternated each minute with "Keep up the good work – you have 4 minutes remaining!" If participants need to stop they can do so, but will be asked every 15 seconds to commence walking again when they feel able.

b) Incremental shuttle walk test (ISWT) [8]. This test is an incremental, progressive, symptom-limited test. The participants will listen to instructions from an audio-tape [8]. They will be asked to walk along a ten-metre track turning around two cones in time with beeps from the tape and to continue until they indicate they need to stop or are unable to keep pace with the beeps. The total distance walked will be recorded.

c) Endurance shuttle walk test (ESWT) [16]. This test is similar to the ISWT in terms of track length and instructions from an audiotape, however, as it is a measure of endurance the participant will be asked to walk at a constant speed for as long as possible (maximum of 20 minutes) following a two minute warm up period. The speed for the ESWT for each participant will be based on the result of his/her ISWT, using the recommended equation [16]. The distance walked during the test will be recorded.

Before and immediately after each walk test the following measurements will be recorded a) oxygen saturation and heart rate using a portable saturation monitor (RAD-5v Masimo Corp, Irvine, CA, USA); b) dyspnoea using the Borg Scale category ratio 0–10 scale [17]. The 6MWT, ISWT and ESWT will be performed twice at each time point to account for a learning effect [18]. Participants will rest for at least 30 minutes between tests or until oxygen saturation, dyspnoea and heart rate return to resting levels. The better distance walked in each test will be recorded for analysis. Testing will be completed over a two-day period at each time point.

2. Quality of life will be measured by:

a) St George's Respiratory Questionnaire (SGRQ) [19]. This is a disease specific, questionnaire shown to be sensitive to changes following pulmonary rehabilitation [19,20]. The SGRQ is a self-administered questionnaire consisting of 50 items. It will be scored from 0 – 100 with zero indicating the best health and 100 the worst. The questionnaire will be explained to the participants. They will be asked to complete it to the best of their ability and to ask for help if they have difficulty understanding the questions.

b) Hospital Anxiety and Depression Scale (HADS) [21]. The HADS has been found to be a reliable instrument for detecting states of depression and anxiety in hospital medical outpatient clinics [21].

3. Lung function will be measured by:

a) Spirometry. This will be performed in accordance with American Thoracic Society standards [22]. Forced expiratory volume in one second (FEV_1_) and forced vital capacity (FVC) will be measured using a mass flow sensor (Sensormedics Vmax 20 Pulmonary Spirometry Instrument; Sensormedics Corporation, Yorba Linda, California, USA). The spirometer will be calibrated immediately before each test using a three litre-calibrating syringe. The highest value for FEV_1 _and FVC after three reproducible trials will be recorded and compared to predicted normal values [23].

b) Lung Volumes. These will be performed in accordance with American Thoracic Society standards [24] using a body plethysmograph (Sensormedics V6200 Autobox Body Plethysmograph; Sensormedics Corporation, Yorba Linda, California, USA). The plethysmograph pressure transducer and the mouth pressure transducer will be calibrated by an automatic internal 50 ml calibration syringe prior to testing. Total lung capacity (TLC) will be determined by summating the best inspiratory capacity (IC) with the mean functional residual capacity (FRC) from three acceptable tests. Residual volume (RV) will be determined by subtracting the best vital capacity (VC) from the calculated TLC value [25]. Results will be compared to predicted normal values for lung volumes [26].

### Statistical analysis

For all outcome measures, the mean difference between the ME group and the control group in the change from baseline to 12 months will be determined and the significance of these mean differences will be tested by repeated measures ANOVA. Significance will be set at p < 0.05. Intention to treat analysis will be used.

### Power calculation

The sample size calculation is based on functional walking capacity as this is the major outcome of the study. Redelmeier and colleagues [27] have reported that in COPD participants, following pulmonary rehabilitation, the minimal clinically important difference is 54 metres. Using this value as the difference in walking distance to be detected between the two groups and taking an effect size of 0.7, it was estimated that the sample size required to achieve an 80% power for a 2-sided test at 5% alpha would be 32 subjects per group. To allow for 15% attrition, 38 subjects will be recruited in each group.

## Discussion

The primary aim of the study is to determine if a once a week supervised, hospital-based exercise plus a home exercise program will maintain functional exercise capacity and quality of life in COPD subjects compared to standard care of unsupervised home exercise, twelve months following the completion of pulmonary rehabilitation. We hypothesize that exercise capacity and quality of life will be better maintained in the ME group compared to the control group.

Pulmonary rehabilitation programs consisting of exercise and education have been shown to improve functional exercise capacity and quality of life [28]. Benefits appear to be maintained up to nine months following pulmonary rehabilitation [6,7] and then begin to decline by twelve months [9]. According to the Survey of Pulmonary Rehabilitation Programs in Australia, following a pulmonary rehabilitation program patients are given advice on home exercise but very few are offered any further form of supervised exercise training [29]. It is important to evaluate if improvements gained from a pulmonary rehabilitation program are able to be maintained in the longer term. There is little scientific information on the relative merit of a supervised, hospital-based exercise program compared to an unsupervised home exercise program in sustaining the benefits of an intensive eight-week pulmonary rehabilitation program in the longer term. Such information is crucial as the goal of pulmonary rehabilitation must be to both maximise and sustain benefits. If greater adherence to an exercise program occurs by supervision, the benefits of a pulmonary rehabilitation program are more likely to be maintained.

This study may have a significant impact on the delivery of health care to people with COPD. If it is shown that supervised maintenance exercise programs ensure the benefits of pulmonary rehabilitation are retained, in terms of exercise capacity and quality of life, then such programs should be routinely instituted.

## Competing interests

The author(s) declare that they have no competing interests.

## Authors' contributions

LMS, JAA and ZJM all contributed to the design of the study, the preparation of the manuscript, and have read and approved the final manuscript.

## Pre-publication history

The pre-publication history for this paper can be accessed here:


